# The Baltimore Academy of Medicine

**Published:** 1886-12

**Authors:** 


### THE BALTIMORE ACADEMY OF MEDICINE.

Officially Reported for the ATLANTA MEDICAL AND SURGICAL JOURNAL.

STATED MEETING, October 19, 1886.

### SUDDEN FAILURE OF HEART'S ACTION THE RESULT OF DIMINUTION OF ATMOSPHERIC PRESSURE.

Dr. Frank Donaldson, jr., referred to a case of sudden failure of heart's action that he had recently seen. The cardiac depression was apparently the result of the diminution in atmospheric pressure that the patient experienced while in a high altitude.

The same sudden heart failure, he observed, is seen as a result of rarefaction of the air in the Pneumatic Cabinet. The approach of the heart failure is indicated by a gradually weakening and irregular action of the pulse.

He thinks the cardiac failure due to a diminution of pressure *upon* the heart, while at the same time the pressure *within* the heart remains the same.

Dr. S. C. Chew has never had his attention called to just such a case, but thinks Dr. Donaldson's explanation a very probable one. He thinks the irregular and tumultuous action of the heart due to a lessened pressure upon the body generally, thus allowing of a universal dilatation of the capillaries.

Dr. F. Donaldson, jr., said that cases of the same nature as the one referred to by him had been seen by other observers—Loomis records 48 of them. Solly has seen a number of these cases in high altitudes, many of whom had never experienced a preëxisting cardiac disturbance previous to their going to these elevations.

### SHOCK CONSEQUENT UPON SURGICAL OPERATIONS.

Dr. J. J. Chisolm said at the last meeting attention had been called to the small amount of shock consequent upon surgical operations upon young children. In his experience old persons stand them as well.

### A CASE OF PLEURISY.

Dr. Henry M. Wilson said he had been in attendance upon a lady for about six weeks or two months. She was very carefully nursed by her sister during this time. One day she called his attention to a slight dyspnœa, and referred to a slight pain in the side, only lasting for a few minutes, that she had felt a few nights previous. Upon examination he found the pleura on that side in a state of inflammation, with its cavity filled with fluid and the heart in consequence displaced. Thought it singular that so extensive an inflammation should have been in progress with so few outward symptoms.

Dr. S. C. Chew said often a less extensive inflammation in this locality gave rise to much more pronounced symptoms than where the process is more extensive. He related a case in his own practice similar in general to that of Dr. Wilson. It was a man, and his pleural cavity became filled with fluid before he was aware of the existence of any trouble whatever of a serious nature.

### PERITONITIS DUE TO PERFORATION OF VERMIFORM APPENDIX.

Dr. Henry Wilson referred to a patient, man, æt. 27 years, whom he had seen. He found him complaining of a not very severe pain over the abdomen. The next morning he was sent for hurriedly and found his patient in collapse, from which he aroused him only with the greatest difficulty. The pain continued without being lessened. He diagnosed peritonitis. The man had had during the previous summer moderate pains over the abdomen at different times. Two weeks after having first seen his patient he died.

Autopsy revealed a swelling containing fluid over the left inguinal ring. It was aspirated and about three pints of fetid pus escaped. Within the abdominal cavity there was a massing together, by means of a fibrous exudation, of the omentum and intestines in such a way as to form, so to speak, a second diaphragm, dividing the cavity into two distinct parts. The pelvic portion was filled with pus.

The vermiform appendix was perforated, and in the most dependent portion of the pelvic cavity there was found a hard mass of fecal matter about the size of a minie-ball. This mass was without a nucleus, as was shown by section made at a later date. He thinks perforation took place at the time of the extreme collapse to which he referred. He referred to a peculiar pain in the penis, that lasted for about half a day, as one of the most prominent symptoms.

### INTUBATION OF THE LARYNX.

Dr. F. Donaldson, jr., recently intubated the larynx of a child with membranous croup. There was apparently a temporary benefit, but the child died in about eight hours.

Dr. S. C. Chew, who had seen Dr. Donaldson's patient, was yesterday called in consultation to see another child suffering from the same trouble, but in a much more precarious condition. The parents of the child refused to permit any operative procedure. Though he has not seen the patient since, he imagines it must be dead. Does not think tracheotomy or intubation of the larynx would have done any good, as the obstruction was less in the larynx than farther down in the trachea.

### ASTIGMATISM IN TWINS.

Dr. J. J. Chisolm has recently seen two cases of interest—two men, twins, æt. nineteen years—each suffering from the same degree of astigmatism and of the same angles.

Two girls, twins, æt. 12 years, each likewise suffering from the same degree of irregular astigmatism and the same angles.

### THE INFLUENCE OF REMOVAL OF ONE OVARY UPON THE SEX OF SUBSEQUENT CHILDREN.

Dr. S. C. Chew spoke of a young lady from whom one of her ovaries had been removed. As she is about to be married, it will be of interest to note the effect upon the sex of her children.

Dr. B. B. Browne said the theory of removal of an ovary affecting the sex of the subsequent children he thought pretty well abandoned, because of women with but a single ovary having given birth to children of different sexes.

Dr. J. J. Chisolm referred to a case of

POISON BY COCAINE.

One year ago he operated upon a man for glaucoma. Recently, while on his way up the bay, the man caught cold, and in consequence there was a conjunctivitis set up. He applied to the inflamed conjunctiva a 5 per cent. solution of nitrate of silver, after first having anæsthetized the eye with cocaine. In half an hour sight was reduced in that eye to such an extent that he barely had perception to light. The man had, in consequence of the instillation of cocaine, an acute traumatic glaucoma.

Eserene was applied to the affected organ, with the result of counteracting the action of the cocaine, and in less than three hours its action was apparent, and in five hours sight in that eye was completely restored. He thinks had eserene not been applied a destructive glaucoma would most certainly have set up. This is the first case of the kind he has seen.

Dr. B. B. Browne has used cocaine with good effect in the vomiting of pregnancy. He administers 10 gtt. of a 2 per cent.

Dr. H. P. C. Wilson exhibited

A NOVEL FORM OF CLINICAL THERMOMETER

that he had recently purchased in London. It is encased in a locket and can be worn upon the watch-chain. It, however, possesses the great disadvantage of not being self-registering.

